# Major response of a peritoneal mesothelioma to nivolumab and ipilimumab: a case report, molecular analysis and review of literature

**DOI:** 10.3389/fonc.2024.1410322

**Published:** 2024-07-18

**Authors:** Marie-Florence Reveneau, Julien Masliah-Planchon, Manuel Fernandez, Abdenour Ouikene, Bernard Dron, Innocenti Dadamessi, Charles Dayen, Lisa Golmard, Bruno Chauffert

**Affiliations:** ^1^ Department of Genetics, Institut Curie, Paris, France; ^2^ Department of Medical Oncology, Saint Quentin Hospital, Saint Quentin, France; ^3^ Department of Radiology, Saint Quentin Hospital, Saint Quentin, France; ^4^ Department of Digestive Surgery, Saint Quentin Hospital, Saint Quentin, France; ^5^ Department of Pneumology, Saint Quentin Hospital, Saint Quentin, France

**Keywords:** peritoneal mesothelioma, nivolumab, ipilimumab, BAP1, PD-L1, molecular stratification, immune checkpoint inhibitors

## Abstract

Malignant peritoneal mesothelioma (MPM) is a rare tumor associated with a poor prognosis and a lack of consensus regarding treatment strategies. While the Checkmate 743 trial demonstrated the superiority of first-line nivolumab and ipilimumab over chemotherapy in malignant pleural mesothelioma (MPlM), few studies have assessed the effectiveness of immunotherapy against MPM, due to its rarity. Here, we report a major and sustained 12-month response in a 74-year-old female patient who received the anti-PD-1 nivolumab and the anti-CTLA4 ipilimumab as first-line therapy for diffuse MPM. PD-L1 was expressed and BAP1 expression was lost, as shown by immunohistochemistry, however the *BAP1* gene was not mutated. Our findings suggest a role for ICI in non-resectable diffuse MPM exhibiting PD-L1 overexpression and loss of BAP1 expression, and instill new hope in their treatment. To our knowledge, this is the second reported case of dual immunotherapy used as first-line in MPM with a major clinical response. To investigate the clinical outcome, we conducted additional molecular analyses of the MPM tumor and we reviewed the literature on immunotherapy in MPM to discuss the role of PD-L1 and BAP1.

## 1 Introduction

Malignant peritoneal mesothelioma (MPM) is a rare tumor with a poor prognosis that develops from the parietal cells of the peritoneum. Its main risk factors are prolonged asbestos exposure and germline pathogenic variants in the *BAP1* tumor suppressor gene (1, 2).

There is no consensus on the management of MPM. When the tumor is resectable, the treatment approach usually relies on cytoreductive surgery with or without hyperthermic intraperitoneal chemotherapy (HIPEC) (3). For advanced or unresectable MPM, systemic chemotherapy with cisplatin and pemetrexed (+/- bevacizumab) is the predominant treatment, as was the case for malignant pleural mesothelioma (MPlM) until 2021 (1, 2, 4). Since then, the combination of nivolumab and ipilimumab has become the standard first-line treatment for MPlM. Indeed, the Checkmate 743 trial demonstrated the superiority of the combination of the two immune checkpoint inhibitors (ICI) anti-PD-1 nivolumab and anti-CTLA4 ipilimumab in the efficacy on overall survival, compared to first-line chemotherapy with cisplatin-pemetrexed in patients with unresectable MPlM (5).

However, despite some studies reporting encouraging results, the efficacy of ICI in MPM has not been formally demonstrated due to the low number of MPM cases in clinical trials, which predominantly include MPlM. The molecular heterogeneity between MPM and MPlM also complicates the interpretation and extrapolation of results (1, 2, 6, 7). Predictive biomarkers for response to immunotherapy in MPM are poorly defined. About 47 to 60% of MPM cases exhibit loss-of-function mutations in *BAP1*, and nearly 50% show overexpression of the PD-L1 protein (6).

BAP1 (BRCA1-Associated Protein 1) is an ubiquitin hydrolase enzyme involved in various pathways including chromatin remodeling and genome integrity maintenance through homologous recombination DNA repair (8, 9). Haploinsufficiency of the *BAP1* gene in MPM has also been associated with strong immunogenicity of the tumor microenvironment and hyperactivation of immune checkpoint receptors PD-1, PD-L1 and CTLA4. Therefore, new studies are crucial to assess whether loss of BAP1 expression and overexpression of PD-L1 are biomarkers for MPM response to ICI (10, 11).

Here, we present the case of a 74-year-old woman diagnosed with diffuse epithelioid-type MPM infiltrating the ileocecal and uterine regions. First-line dual immunotherapy using nivolumab and ipilimumab was used by analogy to MPlM and was efficient.

## 2 Case report

**Figure 1:**
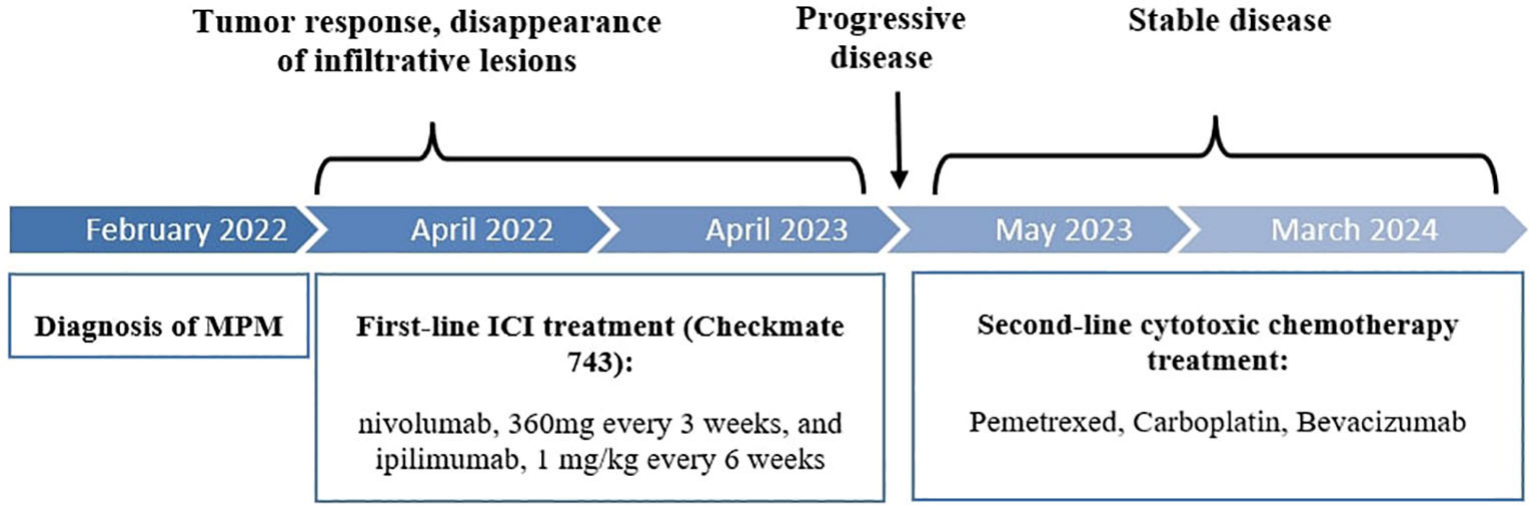
Timeline of the patient’s care course.

**Figure 2:**
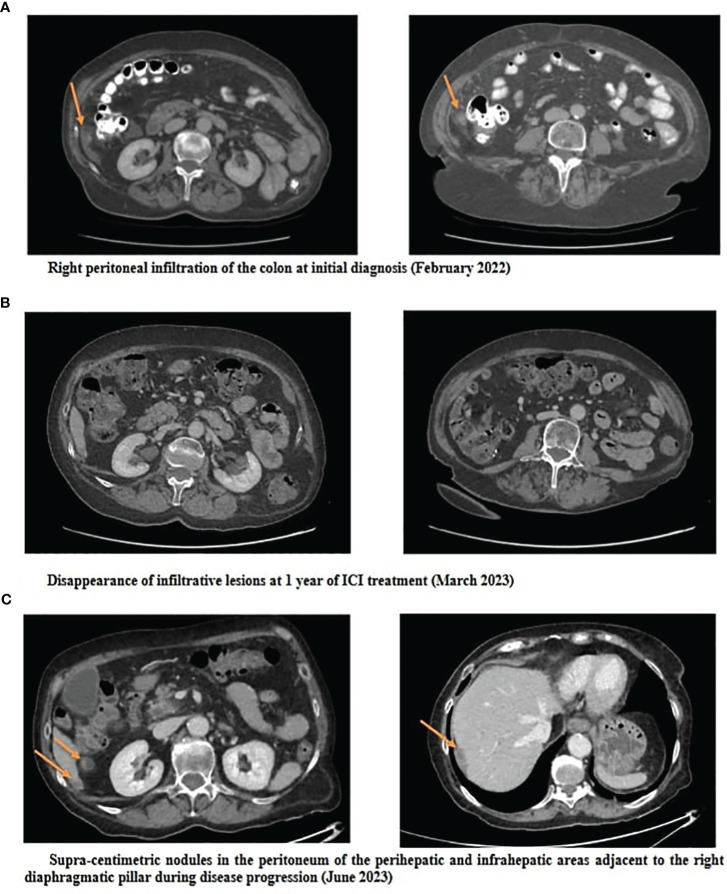
Abdominopelvic CT scans at initial diagnosis **(A)**, at 1 year of ICI treatment **(B)** and at progression **(C)**.

A 74 year-old female patient was referred in February 2022 for weight loss and abdominal pain in the right iliac fossa. Performance status was altered (WHO score 3). Medical history revealed hypertension and a surgically treated syndrome of the pyelo-ureteral junction. There was no personal or family history of cancer, nor any history of exposure to asbestos. Colonoscopy and gastroscopy yielded normal results. Contrast-enhanced CT scan revealed thickening of the peritoneum adjacent to the right colon and cecum, along with infiltration of the right lateral-uterine region and small right pleural effusion (**Figures 1**, **2A**). A laparotomy was conducted, during which biopsies revealed a diffuse infiltrating epithelioid-type malignant peritoneal mesothelioma without possibility of complete resection. Additionally, the surgical treatment was contraindicated due to pleural metastatic involvement. Diagnosis was confirmed by the French National Reference Network MESOPATH. Immunohistochemical staining (IHC) showed the loss of expression of the BAP1 protein and expression of the PD-L1 protein in tumor-infiltrating lymphocytes (TILs) and tumor cells, with a combined positive score (CPS) of 10. Ki67 proliferation index was 5%. Tumor mutational burden (TMB) was low. The treatment decision was based on the first-line treatment of unresectable MPlM, and considering the expression of the PD-L1 protein. After providing clear information to the patient and her husband regarding the off-label use of this treatment for MPM, she consented and signed an informed consent. From April 2022 to April 2023, she received 10 injections of nivolumab, 360 mg every 3 weeks, and ipilimumab, 1 mg/kg every 6 weeks, following the regimen outlined in the Checkmate 743 trial for MPlM. Abdominal pain disappeared and performance status improved, without any significant toxicity. Subsequent CT scans showed a reduction in peritoneal infiltration (**Figure 2B**). However, in May 2023, the patient experienced renewed pain in the right hypochondria. A CT scan revealed new supra-centimetric nodules in the peritoneum, located in the perihepatic and infrahepatic regions, in contact with the right diaphragm, in the pelvis and in the right pleura (**Figure 2C**). Immunotherapy was discontinued and a regimen of pemetrexed, carboplatin and bevacizumab was initiated and led to a sustained major response. In March 2024, 24 months following the initial diagnosis, the patient remained well while receiving a pemetrexed and bevacizumab maintenance therapy.

## 3 Molecular analysis

To investigate the hypothesis explaining the notable clinical response, genomic analyses were conducted on the tumor tissue. Specifically, we focused on commonly mutated tumor suppressor genes in MPM, including *NF2, CDKN2A, CDKN2B, PBRM1, TP53*, *SETD2* and *SETDB1* (12–14). Our examination of these genes in the patient’s tumor did not identify any mutations. Additionally, we sought mutations in oncogenes, such as *KRAS, EGFR, FGFR3, ALK*, which have been documented in rare cases of MPM (7, 13). However, our analysis of these oncogenes in the patient’s tumor did not uncover any mutations.

**Figure 3:**
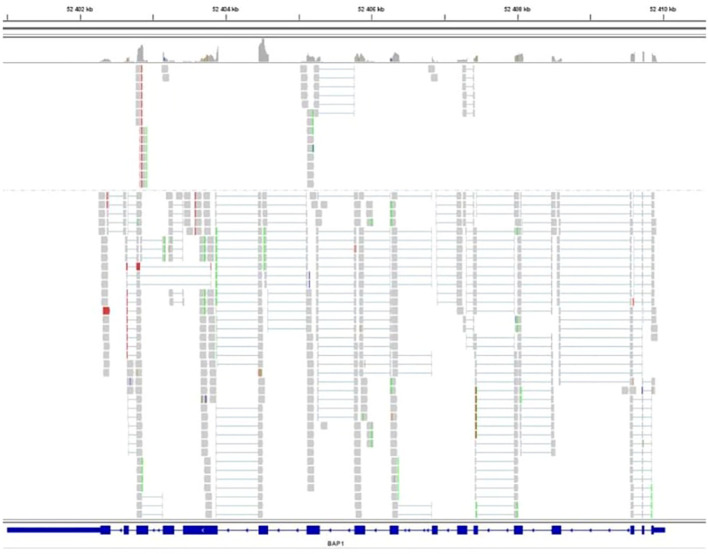
Sequencing of the *BAP1* transcript. *BAP1* exons are represented as solid rectangles. RNA sequence reads are shown in grey and exhibit homology to the reference sequence, indicating no splicing alteration.

The gene sequencing of the tumor unveiled the absence of alterations in the *BAP1* gene despite the loss of protein expression in IHC. Moreover, no modifications were detected in the gene transcript (**Figure 3**).

## 4 Discussion

Here, we present a major and sustained 12-month response of a patient with MPM treated in the first line with nivolumab and ipilumumab. In 2022, Rizzolo et al. reported a near-complete response in a patient with resectable MPM treated in the first line with the combination of nivolumab and ipilimumab before and after surgery, suggesting a role for perioperative use of ICI in operable MPM cases (15). To our knowledge, we present the second reported case of dual immunotherapy used in first line in MPM with a major clinical response. Several studies have reported promising outcomes with the use of ICI in MPM following initial treatment. **Table 1** provides an overview of these studies, confronting the main histological and molecular characteristics, including BAP1 and PD-L1 expression status when available, with clinical outcomes. Currently, a phase II prospective trial is ongoing, focusing on perioperative Nivolumab and Ipilimumab in resectable MPM (Clinical Trials ID: NCT05041062). Interestingly, our patient also presented an ongoing 10 month response to a subsequent chemotherapy regimen by pemetrexed, carboplatin and bevacizumab. The biological basis of this second major response is unknown, but a similar observation has been reported for paclitaxel and platinum salt after ICI failure in head and neck carcinoma (26).

**Table 1:** Overview of ICI in MPM regarding PD-L1 and/or BAP1 status. (A) Case reports.

Case reports	Histological type	PD-L1 status	BAP1 status	Prior surgery	Prior lines of systemic chemotherapy	Immunotherapy	Response under ICI
**Tanaka et al.** (16) **70 y-o man**	Epithelioid	Unknown	Unknown	No	L1: Cisplatin-Pemetrexed	L2: Nivolumab (24 cycles)	Partial response Progression free for 10 months (m) after cessation of ICI*
**Becker et al.** (14) **43 y-o woman** **56 y-o woman** **70 y-o man** **72 y-o man**	Sarcomatoid	IHC: 91%	IHC: positive	Unknown	L1: Cisplatin-Pemetrexed-Bevacizumab L2: Gemcitabine	L3: Nivolumab (13 cycles)	Partial response (PFS: 6m; OS: 8.7m)
	Sarcomatoid	IHC: 100%	IHC: positive	Unknown	L1: Cisplatin-Pemetrexed-Bevacizumab	L2: Nivolumab (4 cycles)	Stable disease (PFS 2.5m; OS 2.7m)
	Epithelioid	IHC: 0%	IHC: LOE SG: 1 pathogenic variant	Unknown	L1: Carboplatin L2: Pemetrexed L3: Gemcitabine	L4: Nivolumab (7 cycles)	Stable disease (PFS 38m; OS 5.2m)
	Epithelioid	Unknown	IHC: positive	Unknown	L1: Cisplatin-Pemetrexed-Bevacizumab	L2: Nivolumab (4 cycles)	Stable disease (PFS 2m; OS 8.1m)
**Foote et al.** (17) **28 y-o man** **61 y-o man**	Epithelioid	Negative	SG: LOF CG: normal	CRS with NIPEC	L1: Cisplatin-Pemtrexed	L2: Association Pembrolizumab + Cisplatin-Pemtrexed	Near-complete response In good health 71m after the diagnosis and 28m after initiation of ICI
	Papillary	Unknown	SG: LOF	2 CRS (1 with HIPEC)	L1: Cisplatin-Pemtrexed	L2: Association Pembrolizumab + chemo	Partial response for 14m. Progression and death 19m after the initial ICI
**Huang et al.** (18) **65 y-o woman**	Uncertain	Unknown	Unknown	HIPEC without CRS	L1: Cisplatin- Pemetrexed	L2: Association ICI (unspecified) + chemo	Progression Death 2m after diagnosis
**Rizzolo et al.** (15) **52 y-o woman**	Epithelioid	Unknown	IHC: LOE GC: normal	CRS	No	L1: Ipilimumab + Nivolumab	Near-complete response for 6m after ICI initiation
**Sugarbaker et al.** (19) **32 y-o woman**	Sarcomatoid-predominant biphasic	Unknown	IHC: LOE	Hysterectomy	L1: Cisplatin-Paclitaxel	L2: Nivolumab	Partial response for 8m until progression**
**Our patient,** **74 y-o woman**	Epithelioid	IHC: positive, CPS 10	IHC: LOE	No	No	L1: Ipilimumab + Nivolumab	Major response for 1y of ICI. Progression. Beginning of pemetrexed, carboplatine and bevacizumab. The patient is in good health at 24m after diagnosis, under pemetrexed and bevacizumab maintenance.

CG, Constitutional Genetics; CRS, Cytoreduction Surgery; CPS, PD-L1 Combined Positive Score; DCR, Disease control rate; HIPEC, Hyperthermic Intra Peritoneal Chemotherapy; ICI, Immune Checkpoint Inhibitor; IHC, Immunohistochemistry; LOE, Loss of Expression; LOF, Loss of Function; m, month(s); NIPEC, Normothermic intra-peritoneal chemotherapy; ORR, Overall Response Rate; OS, Overall Survival; PFS, Progression Free Survival; PR, Partial Response; SG, Somatic Genetics; y, year(s).

*The treatment was interrupted for financial reasons.

**The patient had an initial diagnosis of ovarian cancer. She was therefore treated accordingly in the first line. Then, treatment with Nivolumab was beneficial for 8 months until progression. Subsequently, the patient underwent CRS with HIPEC and NIPEC in addition to Cisplatin, resulting in 5-year disease-free survival.

**Table 1:** (B) Retrospective cohorts and trials.

Retrospective cohorts	Key characteristics	Immunotherapy	Significant results
**Marmarelis et al.** (20) **13 patients**	- Histological types: 70% Epithelioid; 15% Biphasic; 7.7% Sarcomatoid; 7.7% Desmoplastic - Prior Pemetrexed +/- Platinum: 100% patients - PD-L1 status: 23% positive; 31% negative; 46% untested	Pembrolizumab	- ORR: 18% - DCR: 81% - Median PFS: 5.7 months - Median OS: 20.9 months - 3 patients with PFS > 2 years - No difference in PFS based on PD-L1 expression or histology
**Raghav et al.** (21) **29 patients**	- Histological types: 86% Epithelioid; 14% Biphasic or Sarcomatoid - Prior line(s) of chemotherapy: 100% patients - Somatic BAP1 loss: 18%	Ipilimumab + Nivolumab: 20 patients (69%) Single ICI agent: 9 patients (31%)	- ORR: 19.2% among evaluable patients - DCR: 65.4% - Median PFS: 5.5 months - Median OS: 19.1 months - 1 year PFS rate: 14% - 1 year OS: 68% - No difference in ORR between single-agent and dual agent ICI
**Marmarelis et al.** (22) **24 patients**	- Histological types: 75% Epithelioid; 16.6% Biphasic; 4.2% Sarcomatoid; 4.2% Desmoplastic - Prior systemic chemotherapy: 95.8% patients - PD-L1 status: 25% positive; 45.8% negative; 29.2% unknown	Pembrolizumab	- Median PFS: 4.9 months - Median OS: 20.9 months from Pembrolizumab initiation and 81.6 months from initial diagnosis - 3 patients (12.5%) with PFS > 2 years - No association between PR and the presence of a BAP1 alteration, PD-L1 positivity, or non-epithelioid histology - PFS did not differ based on PD-L1 status
Phase II single-arm trials	Key characteristics	Immunotherapy	Significant results
**Desai et al.** (23) **8 patients**	**Refractory MPM** (The trial included 8 MPM and 56 MPlM) - All patients received prior systemic chemotherapy - PD-L1 and BAP1 status: unknown	Pembrolizumab	- RR: MPM 20% (vs MPlM 12.5%) - ORR: MPM 12.5% (vs MPlM 20%) - Regardless of the site: no correlation between PD-L1 and RR; trend to higher RR in PD-L1 ≥1%
**Raghav et al.** (24) **20 patients**	**Relapsed/refractory and unresectable MPM** - Histological types: Epithelioid (90%); Biphasic (10%) - Prior line(s) of chemotherapy: 100% patients - PD-L1 status: 31% negative; 69% positive	Atezolizumab + anti-VEGF Bevacizumab	- ORR: 40% - 1 year OS: 85% - 1 year PFS: 61% - Median PFS: 17.6 months - Responses occurred in PD-L1 positive and negative patients with a trend towards a higher RR positive PD-L1

As an indication, the clinical outcomes of MPM patients treated with standard treatments are as follows: CRS with HIPEC: survival between 34 and 92 month; 5-year OS between 40–70%; 10-year survival 26%; Systemic Pemetrexed-Cisplatin: ORR 26%, SDR 45%, DCR 71% (2, 25).

DCR, Disease Control Rate; ORR, Overall Response Rate; OS, Overall Survival; PFS, Progression-Free Survival; PR, Partial Response; RR, Response Rate; SDR, Stable Disease Rate.

NB 1: Regarding studies that included both MPM and MPlM, only one with sufficient power for MPM has been reported.

NB 2: A phase II prospective trial is ongoing, focusing on perioperative Nivolumab and Ipilimumab in resectable MPM (Clinical Trials ID: NCT05041062).

In some solid tumors, such as advanced clear cell renal cell carcinoma, *BAP1* alterations have been reported as a significant predictor of the immune microenvironment and have been associated with a longer progression-free survival (PFS) in patients when treated with ICI (27). *BAP1*-altered MPM are associated with a more inflammatory tumor microenvironment and seem to constitute a distinct immunogenic class, with possible implications for immunotherapeutic response (10, 11, 28, 29). In their study, Shrestha et al. found that *BAP1*-altered MPM had higher levels of immune checkpoint receptors (PD-1, PD-L1, and CTLA4) with higher cytokine secretion and an increased recruitment of T lymphocytes, leading to genomic instability and a DNA repair defect, compared to wild-type *BAP1* MPM (10). In the reported case, the loss of BAP1 expression in IHC was identified, with no corresponding alteration observed in the *BAP1* gene or its transcript. The absence of identified mutations in the *BAP1* gene despite the loss of the protein may be explained by alterations in the *BAP1* regulatory regions, such as the promoter and introns, through complex structural variants like promoter deletions that were not detected by the panel. Inactivation of BAP1 expression by *BAP1* methylation, as has been observed in some uveal melanoma cases, could also be a contributing factor (30, 31). Other epigenomic alterations have been suggested to contribute to carcinogenesis in MPM (13). Indeed, Bozzi et al. have described alterations of epigenetic regulator genes that may affect BAP1 expression, such as *EZH2*, reporting a correlation between strong expression of *EZH2* and the loss of BAP1 in MPM samples (13, 32). *EZH2* mutations were specifically investigated in our patient’s tumor and were not detected. However, for investigating a potential correlation between EZH2 and BAP1 expression, analyzing EZH2 protein levels would provide more informative insights, given that its overexpression is primarily influenced by epigenetic, transcriptional, and post-transcriptional alterations. Loss of the BAP1 protein, despite the absence of alterations in the *BAP1* gene and its transcript, has previously been reported in mesotheliomas and other *BAP1*-related neoplasms, including clear cell renal carcinoma and uveal melanoma. Proportions of IHC-negative cases associated with a wild-type *BAP1* gene have ranged from 11% to 25% among the samples (33–35). Bott et al. identified such MPlM cases, characterized by normal BAP1 mRNA expression, suggesting the possibility of post-translational dysregulations leading to its loss-of-function (33, 36). Dysregulation of ubiquitination, which plays a role in the metabolic reprogramming of cancer cells, might be one such mechanism and has also been reported in certain cases of MPM (13). Regarding the genomic profile of our patient’s tumor, the absence of identified mutations in *BAP1*, coupled with the patient’s solitary neoplasm and the absence of a family history of *BAP1*-related tumors, make it highly unlikely that the origin of her tumor was constitutional. It is more probable that it resulted from acquired genomic and/or post-translational events leading to the loss of BAP1 expression, subsequent DNA repair defects, and genomic instability leading to a robust immune response in the microenvironment through the recruitment of cytokines and T lymphocytes. In this context, our patient’s MPM might align with a specific subgroup identified by Hiltbrunner et al. (*BAP1* alteration without *CDKN2A/B* alteration) associated with a better prognosis (7). These findings are also consistent with the results of Osmanbeyoglu et al., who identified a distinct subgroup of MPlM characterized by specific immunogenicity, a PD-L1 response signature, and longer survival in patients with altered *BAP1* alone, without abnormalities in *CDKN2A/B* or *NF2* genes, suggesting that *BAP1* loss alone could serve as a candidate marker for ICI therapy (29).

Our patient’s tumor had a low TMB, which is common in MPM (6). However, high TMB in some solid cancers (e.g., melanoma) is a biomarker of a favorable response to ICI through accumulation of neo-antigens from tumor mutations (37). Tumors with a low TMB can still trigger robust immune responses if their mutations lead to the expression of immunogenic neo-antigens. Solid tumor immunogenicity also relies on factors independent of mutational burden, including T-cell migration, PD-1 expression, cytokine balance, metabolic regulation, and *BAP1* gene mutations in MPM (38–40). Hence, when considering ICI treatment for MPM, the presence of a low TMB should not preclude the assessment of PD-L1 and BAP1 expression status, which seem to be better indicators of lymphocyte infiltration than TMB. Moreover, in the absence of an increase in TMB, mutations of the *SETDB1* gene in MPM (which have not been found in our patient’s tumor) also appear to be a potential marker of sensitivity to ICI, as observed in the patients reported by Becker et al. (14).

Significant clinical responses have been observed in patients with BAP1 loss and negative PD-L1 expression, as well as *vice versa*. Foote et al. reported a quasi-complete response in a 28-year-old MPM patient treated with Pembrolizumab and Cisplatin-Pemetrexed, despite negative PD-L1 expression (17). Phase II single-arm trials by Raghav et al. and Desai et al. also showed significant clinical responses to PD-L1 or PD-1 inhibitors, regardless of PD-L1 status, although PD-L1 positive patients tended to have a better response rate (21, 23). Similarly, retrospective cohort studies by Marmarelis et al. found ICI responses across various PD-L1 statuses (20, 22). Checkmate 743 trial results in MPlM did not find a significant difference in survival based on PD-L1 status in patients treated with nivolumab-ipilimumab (5). The Raghav et al. trial showed promising results with the Atezolizumab-Bevacizumab “AtezoBev” combination therapy in both PD-L1 positive and negative patients, with slightly higher response rates in PD-L1 positive patients (24). Ongoing phase II multi-arm stratified trial MiST (Mesothelioma Stratified Therapy, ClinicalTrials.gov Identifier: NCT03654833) is currently investigating the AtezoBev combination in MPlM with PD-L1 overexpression. These observations led to the hypothesis that a significant immune response may be triggered by anti-PD-1/anti-PD-L1 agents, even in cases with PD-L1-negative tumors. In this regard, Foote et al. documented the emergence of significant tumor immune mobilization mediated by CD4 and CD8+ T lymphocytes following the initiation of Pembrolizumab in their patient, resulting in near-complete response of their MPM, while maintaining a negative PD-L1 status (17).

Taken together, these observations underscore the importance of integrating multiple biomarkers to refine the predictions of response to ICI and to refine molecular signatures associated with an ICI response in MPM. In a 3-year follow-up study of the Checkmate 743 trial, Peters et al. tested a four-gene inflammatory signature (based on mRNA expressions of CD8A, STAT1, LAG3 and PD-L1), with a high score correlating with improved survival in patients treated with the nivolumab-ipilimumab combination (41–44). Further studies are needed to determine whether this signature could serve as a biomarker for ICI response in MPM. In the ongoing multi-arm stratified phase II trial MiST (ClinicalTrials.gov Identifier: NCT03654833) focusing on MPlM, patients with *BRCA1* or *BAP1* alterations receive a Poly (ADP-ribose) polymerase (PARP) inhibitor, while patients with positive PD-L1 expression receive the “AtezoBev” combination therapy. However, in their phase II trial, Ghafoor et al. found only limited efficacy of olaparib in 23 patients with MPlM and MPM harboring a *BAP1* mutation (45). The effectiveness of anti-PARP therapy in *BAP1*-mutated MPM remains uncertain, and further studies involving a larger cohort of patients are warranted.

## 5 Conclusion

In conclusion, we present a major and sustained 1-year response in a non-resectable diffuse MPM treated with first-line dual ipilimumab and nivolumab. Our case suggests a potential role for ICI in non-resectable diffuse MPM cases exhibiting PD-L1 overexpression and loss of BAP1 expression, and instills new hope in their treatment. However, a cautious interpretation of these findings is needed, and response rates should not be extrapolated from this case report, as there is significant publication bias in this field. Refinement of molecular classification and identification of potential biomarkers of ICI response in MPM, including PD-L1 and BAP1 status, are imperative for patient stratification and to guide therapeutic decision-making.

## Data availability statement

The original contributions presented in the study are included in the article and/or can be inquiried about directly from the corresponding author.

## Ethics statement

The studies involving humans were approved by Ethics committee of the Saint Quentin Hospital, Saint Quentin, France. The studies were conducted in accordance with the local legislation and institutional requirements. The participants provided their written informed consent to participate in this study. Written informed consent was obtained from the individual(s) for the publication of any potentially identifiable images or data included in this article.

## Author contributions

M-FR: Writing – original draft. JM-P: Writing – review & editing. MF: Writing – review & editing. AO: Writing – review & editing. BD: Writing – review & editing. ID: Writing – review & editing. CD: Writing – review & editing. LG: Writing – review & editing. BC: Supervision, Writing – review & editing.
