# Supplementary material for: Major response of a peritoneal mesothelioma to nivolumab and ipilimumab: a case report, molecular analysis and review of literature
Source: Front Oncol. 2024 Jul 18;14:1410322. doi: 10.3389/fonc.2024.1410322 (PMC11291227; doi:10.3389/fonc.2024.1410322)
Supplement: Supplementary file 1 [file DataSheet_1.docx]

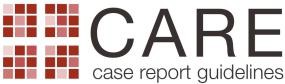
CARE Checklist of information to include when writing a case report
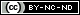


**Topic Item Checklist item description Reported on Line**

**Title 1** The diagnosis or intervention of primary focus followed by the words “case report”

**Key Words 2** 2 to 5 key words that identify diagnoses or interventions in this case report, including "case report"

**Abstract**

**(no references)**

**3a** Introduction: What is unique about this case and what does it add to the scientific literature? __

**3b** Main symptoms and/or important clinical findings

**3c** The main diagnoses, therapeutic interventions, and outcomes

**3d** Conclusion—What is the main “take-away” lesson(s) from this case?

**Introduction 4** One or two paragraphs summarizing why this case is unique (**may include** reference**s**)

**Patient Information 5a** De-identified patient specific information

**5b** Primary concerns and symptoms of the patient

**5c** Medical, family, and psycho-social history including relevant genetic information

**5d** Relevant past interventions with outcomes

**Clinical Findings**

**Timeline**

**Diagnostic Assessment**

**Therapeutic Intervention**

**Follow-up and Outcomes**

1. Describe significant physical examination (PE) and important clinical findings
2. Historical and current information from this episode of care organized as a timeline

**8a** Diagnostic testing (such as PE, laboratory testing, imaging, surveys).

**8b** Diagnostic challenges (such as access to testing, financial, or cultural)

**8c** Diagnosis (including other diagnoses considered)

**8d** Prognosis (such as staging in oncology) where applicable

**9a** Types of therapeutic intervention (such as pharmacologic, surgical, preventive, self-care)

**9b** Administration of therapeutic intervention (such as dosage, strength, duration)

**9c** Changes in therapeutic intervention (with rationale)

**10a** Clinician and patient-assessed outcomes (if available)

**10b** Important follow-up diagnostic and other test results

**10c** Intervention adherence and tolerability (How was this assessed?)

**10d** Adverse and unanticipated events

**Discussion 11a** A scientific discussion of the strengths AND limitations associated with this case report

**11b** Discussion of the relevant medical literature **with references** ___________________

**11c** The scientific rationale for any conclusions (including assessment of possible causes)

**11d** The primary “take-away” lessons of this case report (without references) in a one paragraph conclusion

**Patient Perspective 12** The patient should share their perspective in one to two paragraphs on the treatment(s) they received

**Informed Consent 13** Did the patient give informed consent? Please provide if requested **Yes**
